# Targeted metabolomic analysis in Parkinson’s disease brain frontal cortex and putamen with relation to cognitive impairment

**DOI:** 10.1038/s41531-023-00531-y

**Published:** 2023-06-03

**Authors:** Karel Kalecký, Teodoro Bottiglieri

**Affiliations:** 1 Institute of Biomedical Studies, Baylor University, Waco, TX 76712 USA; 2 Center of Metabolomics, Institute of Metabolic Disease, Baylor Scott & White Research Institute, Dallas, TX 75204 USA

**Keywords:** Metabolomics, Diagnostic markers, Dementia, Parkinson's disease

## Abstract

We performed liquid chromatography tandem mass spectrometry analysis with the targeted metabolomic kit Biocrates MxP Quant 500, in human brain cortex (Brodmann area 9) and putamen, to reveal metabolic changes characteristic of Parkinson’s disease (PD) and PD-related cognitive decline. This case-control study involved 101 subjects (33 PD without dementia, 32 PD with dementia (cortex only), 36 controls). We found changes associated with PD, cognitive status, levodopa levels, and disease progression. The affected pathways include neurotransmitters, bile acids, homocysteine metabolism, amino acids, TCA cycle, polyamines, β-alanine metabolism, fatty acids, acylcarnitines, ceramides, phosphatidylcholines, and several microbiome-derived metabolites. Previously reported levodopa-related homocysteine accumulation in cortex still best explains the dementia status in PD, which can be modified by dietary supplementation. Further investigation is needed to reveal the exact mechanisms behind this pathological change.

## Introduction

More than 6 million people worldwide live with Parkinson’s disease (PD), the second most prevalent neurodegenerative disorder, and the number is on the rise^1^. Progressive death of dopaminergic neurons connecting substantia nigra and putamen, accompanied by accumulation of α-synuclein aggregates called Lewy bodies, leads to gradual loss of motor control. The quality of life can be further reduced by developing dementia, which occurs up to 6 times more often among PD patients^2^. Pure PD dementia is histopathologically different from Alzheimer’s disease dementia although both conditions can develop together, resulting in a mixed pathology^3^.

There is no known cure for PD. At best, the severity of motor symptoms can be reduced by providing the brain with exogenous L-3,4-dihydroxyphenylalanine (DOPA), also referred to as levodopa in the context of the medication, which passes the blood-brain barrier and is converted into dopamine. Levodopa is often sold as mixture with N-amino-α-methyl-3-hydroxy-L-tyrosine monohydrate (carbidopa), which inhibits amino acid decarboxylase thereby reducing peripheral metabolism of levodopa, so that more of the drug can reach the brain. Unfortunately, the treatment effectivity subsides over time, resulting in shorter therapeutic windows of symptom reduction and more frequent side effects such as dyskinesia.

PD is a complex disease. Multiple genetic and environmental factors contribute to the etiology^4^ although not all aspects have been fully elucidated. Parkinson’s disease dementia (PD-D) seems to be even more elusive^5^. Further understanding of the underlying mechanisms is needed to identify reliable biomarkers for early detection and intervention.

On a higher level, the quality of intracellular processes and homeostasis is reflected in metabolism, which represents the functional part of cells and the interplay between tissues. Recent methodological developments in metabolomics have allowed quantitative investigation to be performed across numerous metabolic pathways simultaneously. To date, only few metabolomic studies have been performed directly in PD brain tissue. Some of them measured a single metabolite^6–8^, single pathway^9^, while others focused only on lipids^10–13^. The findings include decreased glutathione^6^, pantothenic acid^7^, increased urea^8^; increased sphingomyelins, ceramides, oxysterols, cholesterol, and altered glycerophospholipids in cortex^10^. In addition, reports indicate increased diacylglycerols, decreased ceramides, and glycerophospholipids in cortex^11^; decreased sphingomyelins and phosphatidylinositol in putamen^12^; and decreased ceramides and increased sulfatide in putamen^13^. None of these studies included more than 15 PD cases, only one corrected for multiple hypothesis testing^10^, and only two had post-mortem collection intervals with differences between samples less than 1 day^6,12^. Larger high-quality metabolomic studies are clearly needed.

We previously performed^14^ a metabolomic study in PD brain tissue that was focused on a single metabolic pathway, specifically one-carbon metabolism. There, we identified homocysteine (Hcy) elevation in frontal cortex with acute levodopa presence as the most characteristic sign of dementia among PD subjects that could not be explained by medication dosage or disease progression. Since the involvement of Hcy in dementia is well established^15^ as well as the potential of levodopa to generate Hcy through its metabolism by catechol-O-methyltransferase (COMT), we provided strong evidence for what has long been suspected^16–19^: the importance of the direct involvement of levodopa in dementia in susceptible individuals.

In the current case-control study, we performed a broad explorative targeted metabolomic analysis in human frontal cortex (Brodmann area 9) and putamen, encompassing the main metabolic pathways as well as certain lipid classes, to better understand brain metabolic changes associated with PD at various stages of cognitive impairment and the effect of levodopa medication.

## Results

### Metabolic differences between controls and PD subjects in relation to cognitive impairment

**Fig. 1:**
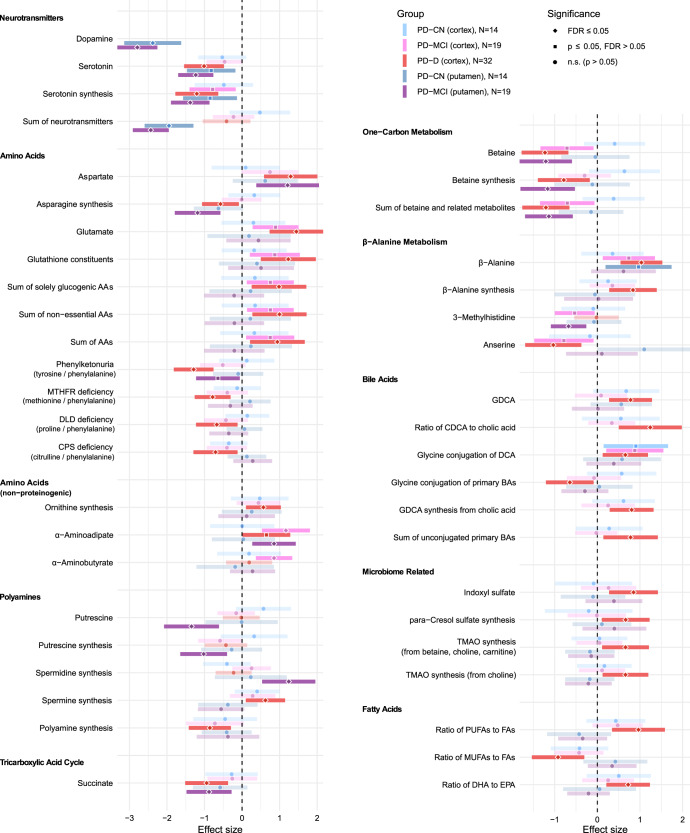
Metabolic differences between subject groups in liquid chromatography experiment. Forest plot for differentially detected metabolites and metabolic indicators in the LC part with regression coefficients for individual PD groups (CN Cognitively normal, MCI – with mild cognitive impairment, D – with dementia of non-Alzheimer’s type) and brain areas. The “N” in the legend denotes the number of samples effectively used to estimate each regression parameter. Values are normalized regression coefficients (depicted as the central points; the shape reflects the significance) with 95% confidence intervals (horizontal range lines; lower opacity for non-significant coefficients). The dashed vertical black line represents a zero effect, i.e. equivalent to controls (for a given brain region). Every analyte listed is statistically significant (FDR ≤ 0.05) for one of the PD groups considered separately or all PD subjects (for a given brain region) considered together. The regression models included covariates as detailed in the Methods.

**Fig. 2:**
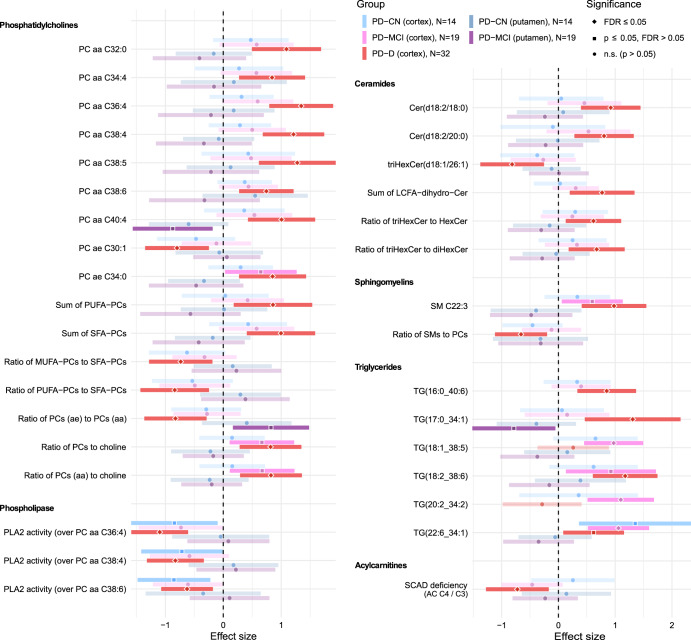
Metabolic differences between subject groups in flow-injection experiment. Forest plot for differentially detected metabolites and metabolic indicators in the FIA part with regression coefficients for individual PD groups (CN – cognitively normal; MCI – with mild cognitive impairment; D – with dementia of non-Alzheimer’s type) and brain areas. The “N” in the legend denotes the number of samples effectively used to estimate each regression parameter. Values are normalized regression coefficients (depicted as the central points; the shape reflects the significance) with 95% confidence intervals (horizontal range lines; lower opacity for non-significant coefficients). The dashed vertical black line represents a zero effect, i.e., equivalent to controls (for a given brain region). Every analyte listed is statistically significant (FDR ≤ 0.05) for one of the PD groups considered separately or all PD subjects (for a given brain region) considered together. The regression models included covariates as detailed in the Methods.

Through a series of linear regression models with covariates adjusting for potential confounding factors (see Methods), we found multiple areas of metabolism different in PD, especially in the groups with cognitive impairment (PD-CI; encompasses both mild cognitive impairment and dementia, which was confirmed as non-Alzheimer’s dementia – see Methods, section Study subjects). Statistically significant results are summarized in Fig. 1 for liquid chromatography (LC) part (small molecules and free fatty acids) and Fig. 2 for flow-injection analysis (FIA) part (complex lipids). All results are listed in detail in Supplementary File 1. Changes in metabolic indicators depend on the context of effects of their constituent metabolites and are mentioned where relevant. Some changes seem to be related to levodopa cycle and will be described in the next section.

The largest difference between PD and controls is in low concentrations of dopamine in PD putamen (Fig. 1), which reflects the pathology definition. Another impacted neurotransmitter is serotonin, visibly decreased in both brain regions in PD. Indicators of phospholipase A2 activity are downregulated in PD cortex (Fig. 2). We detected several bile acids (BAs) in brain tissue including the metabolic indicator of glycine conjugation of deoxycholic acid (DCA) to form glycodeoxycholic acid (GDCA) that was significantly increased in PD cortex, with a similar but non-significant trend in putamen.

Other changes from controls seem to be confined to PD-CI subjects: aspartate and α-aminoadipate are elevated in both brain regions, while betaine (trimethylglycine) is decreased (Fig. 1). Glutamate is elevated in PD-CI groups only in cortex, whereas the polyamine putrescine shows a significant decrease in putamen (Figs. 1, 2). Indicator of ornithine synthesis is higher in PD-D, less significantly in cortex of other PD groups, compared to controls. Several metabolic indicators containing phenylalanine are dysregulated in PD-D with a similar but less significant trend in PD subjects with mild cognitive impairment (PD-MCI). Succinate is downregulated in PD-D in cortex and PD-MCI putamen. α-Aminobutyrate is increased only in PD-MCI cortex.

The decreased indicator of glycine conjugation of primary BAs in PD-D (Fig. 1) is confounded by PD duration as explained further in the section Metabolic associations with disease progression scores.

### Levodopa impact on metabolism and its distinctive effect in PD-D

**Fig. 3:**
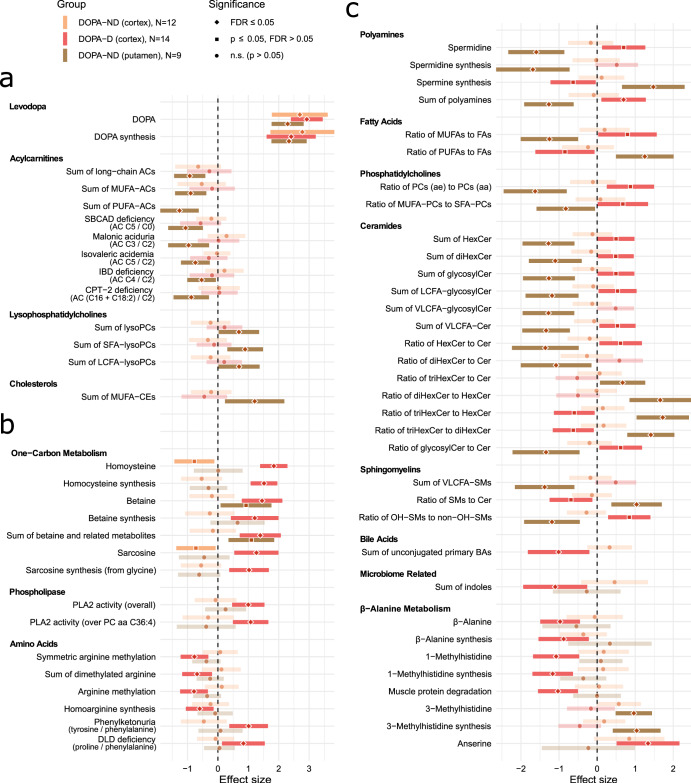
Effect of acute levodopa medication presence on metabolism in PD groups. Forest plot for differentially detected metabolites (LC only) and metabolic indicators (both LC and FIA) with regression coefficients for the acute levodopa presence in PD in interaction with dementia status (ND – no dementia; D – with dementia of non-Alzheimer’s type) and brain areas. These effects are additive to the PD group effects (Figs. 1 and 2) for subjects with acute levodopa presence, as the regression parameters were a part of the same linear regression models. The “N” in the legend denotes the number of samples effectively used to estimate each regression parameter. Values are normalized regression coefficients (depicted as the central points; the shape reflects the significance) with 95% confidence intervals (horizontal range lines; lower opacity for non-significant coefficients). The dashed vertical black line represents a zero effect, i.e. equivalent to PD subjects with physiological DOPA levels (in a given group). Every analyte listed is statistically significant (FDR ≤ 0.05) for levodopa medication in putamen or cortex in interaction with dementia status. Pathways are grouped by similarity in the levodopa effect: (**a**) in all samples or putamen only, (**b**) in PD with dementia cortex or similarly in putamen, and (**c**) in PD with dementia cortex and differently in putamen (upon interpretation with Figs. 1 and 2). The regression models included covariates as detailed in the Methods.

The effect of levodopa-carbidopa medication (on top of the PD subgroup effects in the linear regression sense) appears rather complex. Statistically significant changes with acute levodopa presence (L+), which we define as elevated DOPA levels (see Methods), in combination with the dementia status, are depicted in Fig. 3 (see Supplementary Fig. 1 for individual lipid species) and listed in detail in Supplementary File 1. However, to fully elucidate the metabolic effects, we need to consider results for the effect of PD (Figs. 1 and 2) as well.

The largest discovered levodopa-related change is high concentration of DOPA (Fig. 3a), which reflects our definition of L+ subjects. Another change is in long-chain fatty acid (LCFA) acylcarnitines (ACs), which are decreased in putamen, showing a similar but non-significant trend in cortex. In putamen, we also observe increased lysophosphatidylcholines (LPCs) and monounsaturated fatty acid (MUFA) cholesteryl esters (CEs).

There is a large increase in Hcy specifically in PD-D (Fig. 3b, Supplementary Fig. 2), which we previously reported^14^. This is the most distinguishing alteration that we found related to dementia status in PD, reaching area under the receiver operating characteristic curve (AUC) 89% (95% confidence interval (CI_95_) 75–100%) among L+ subjects (Supplementary Fig. 2). Two other metabolites related to one-carbon metabolism, betaine and sarcosine, follow the Hcy increase (Fig. 3b). In PD-D, levodopa is further associated with elevated phospholipase A2 (PLA2) activity and altered arginine metabolism, as evident by decreased indicators of homoarginine synthesis and symmetric dimethylated arginine (SDMA) methylation (Fig. 3b).

**Fig. 4:**
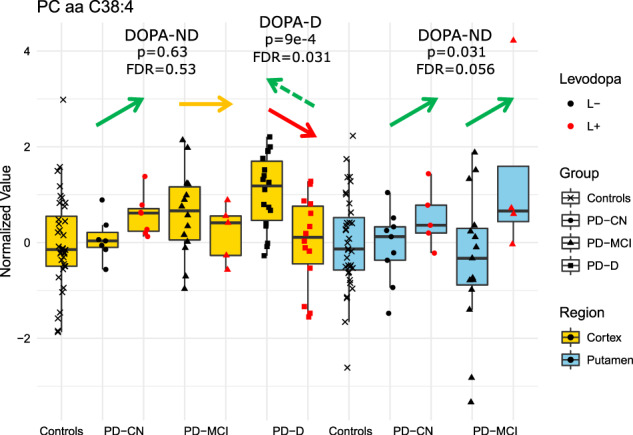
Example of phase delay in levodopa effect in PD with dementia. In this box plot (center line – median; box limits – upper and lower quartiles; whiskers – 1.5× interquartile range), depicted across subject groups (CN – cognitively normal; MCI – with mild cognitive impairment; D – with dementia of non-Alzheimer’s type) in dependence on the acute levodopa medication presence (L+; red points) or physiological range (L−; black points), we show an example of a metabolite (phosphatidylcholine PC aa C38:4; normalized values) with a seemingly opposite levodopa effect in PD-D subjects (decreasing red arrow). However, the values do not go in the opposite direction (below the baseline), but rather the same change happens in the opposite levodopa state (increase in L- instead of a change in L+), suggesting a delay in the levodopa-induced effect in PD-D cortex, potentially starting to appear in PD-MCI cortex. Similar patterns were observed across multiple metabolic classes. Statistical significance of regression coefficients for acute levodopa presence (from the main regression analysis, which includes covariates) is provided.

For several areas of metabolism, levodopa has seemingly opposite effects in putamen and in cortex of PD-D (Fig. 3c). In fact, L+ changes observed in putamen, not present in the state of physiological DOPA levels (L−), well correspond to L- changes in PD-D, which disappear in L+, hence seeing the opposite direction for the levodopa effect (Figs. 1, 2, 3c). Therefore, what we see rather appears to be a delayed effect (or side effect) of the medication on metabolism in PD-D. This behavior, an example of which is captured in Fig. 4, is significant (for both L+ PD putamen and L- PD-D cortex) for decrease in spermidine and several lipid related indicators: decreased ratio of MUFAs compared to polyunsaturated fatty acids (PUFAs), decreased ratio of MUFA phosphatidylcholines (PCs) compared to saturated fatty acid (SFA) PCs, decreased ratio of acyl-alkyl PCs compared to diacyl PCs, and decreased ratios of simpler GCs to complex GCs. Certain indicators related to sphingolipids show the same trend with significant changes in L+ PD putamen and less significant opposite changes (FDR > 0.05, but *p* ≤ 0.05) in L+ PD-D cortex: decreased glycosylceramides (GCs; ceramides with any glycosidic bond), decreased ratio of simpler GCs to ceramides, but increased ratio of trihexosylceramides and SMs to ceramides, and decreased ratio of hydroxylated SMs to non-hydroxylated SMs. Certain triglycerides (TGs) and with lower significance BA indicators exhibit similar patterns.

Additionally, there are metabolites dysregulated in a similar fashion in L- PD-D (Fig. 1): increased indoxyl sulfoxide, and indicators of synthesis of para-cresol sulfoxide and trimethylamine N-oxide. These trends are apparent in L+ PD-MCI cortex as well, but do not reach statistical significance.

Interestingly, β-alanine metabolism is more dysregulated in L- and normalized in L+ (Figs. 1, 3c). The dysregulation consists of increasing β-alanine almost universally, but differs in other parts of the pathway: anserine decreases in cortex of PD-CI only, 3-methylhistidine (π-methylhistidine) slightly fluctuates, especially in non-demented PD subjects (PD-ND), while 1-methylhistidine (τ-methylhistidine) increases in PD-D.

### Affected metabolic pathways based on KEGG database

We also looked at the differential analysis results for each PD group in terms of metabolic set analysis using Kyoto Encyclopedia of Genes and Genomes (KEGG) pathways^20^ for which we were able to map at least 4 metabolites. The results mostly overlap with the findings for individual metabolites. The detected changes (Table 1) are confined mainly to PD-CI and are potentially affected by the putative delayed levodopa medication effects.

**Table 1:**
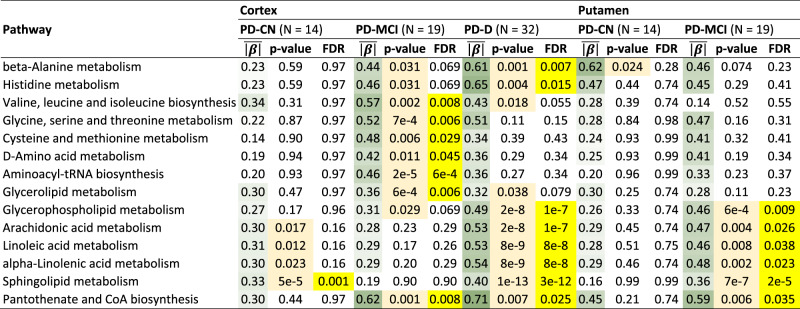
Metabolic set analysis in PD cognitive groups using KEGG pathways.

KEGG pathway metabolic set statistics with statistically significant difference (FDR ≤ 0.05) for at least one of the PD groups as compared to controls. Computed from the main regression models with covariates as detailed in the Methods. Yellow: FDR ≤ 0.05, light orange: *p*-value ≤ 0.05, green: average absolute effect ($\bar{\left|\beta \right|}$) – the darker the color, the higher the value.

*CN* Cognitively normal, *CoA* Coenzyme A, *D* Dementia (non-Alzheimer’s type), *FDR* False discovery rate, *MCI* Mild cognitive impairment, *PD* Parkinson’s disease.

Besides β-alanine, histidine, cysteine and methionine, and D-amino acid metabolism, altered in PD-CI cortex and with a similar trend in putamen, we also see disrupted branched-chain amino acid biosynthesis in PD-CI cortex (Table 1). Glycine, serine and threonine metabolism is impacted in PD-MCI cortex, with a similar tendency in other cognitively impaired groups, alongside aminoacyl-tRNA biosynthesis. This suggests that the amino acid metabolism is affected more than discovered from individual metabolites.

Among lipids, glycerolipid and glycerophospholipid metabolism are altered in PD-CI, while several PUFA pathways (arachidonic acid, linoleic acid, and α-linolenic acid metabolism) are mainly affected in PD-D and in PD-MCI putamen but not PD-MCI cortex (Table 1). Sphingolipid metabolism is similar, additionally showing a significant difference in PD-CN cortex.

Interestingly, we see pantothenate and coenzyme A biosynthesis affected in PD-CI (Table 1).

### Metabolic associations with disease progression scores

Next, we explored associations between analytes (metabolites and metabolic indicators) and measures of disease progression and cognitive impairment, including both clinical and histopathological scores, among PD subjects. Covariates were included as previously, only the acute levodopa status was omitted for its complicated relationship with PD cognitive subgroups, which were not considered in this part and all PD subjects were analyzed together. The significant results are listed in Table 2 (see Supplementary File 2 for complete results) and most of them relate to putamen. The less significant brain region follows the same trends with only several exceptions.

**Table 2:**
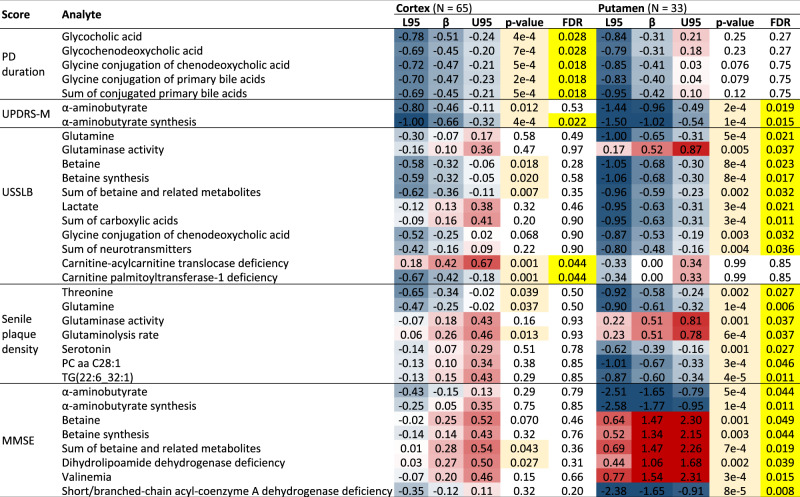
Metabolic associations with progression scores in PD subjects.

Statistically significant (FDR ≤ 0.05) associations with several progression scores in either brain region among PD subjects. UPDRS-M score, measured in off-levodopa state, is known for 57% of PD subjects. MMSE score is known for 88% of PD subjects. Yellow: FDR ≤ 0.05, light orange: *p*-value ≤ 0.05, red/blue: positive/negative effect (the darker the color, the higher the magnitude) with two-way standardization.

*aa* diacyl, *β* Effect size (regression coefficient), *L95* Lower bound of 95% confidence interval, *MMSE* Mini-Mental State Examination, *PC* Phosphatidylcholine, *PD* Parkinson’s disease, *TG* Triglyceride, *U95* Upper bound of 95% confidence interval, *UPDRS-M* Motor section of the Unified Parkinson’s Disease Rating Scale, *USSLB* Unified Staging System for Lewy Body Disorders.

In cortex, duration of PD since diagnosis is associated with decreasing glycine conjugation of primary bile acids to form glycocholic and glycochenodeoxycholic acids (Table 2). A similar although weaker association with glycine conjugation of primary BAs was found in connection with PD-D, and given that the PD-D group also has longer PD duration compared to other groups, we suspected a confounding effect. Indeed, further exploration revealed that this association for PD-D is confounded by the diagnosis duration: Diagnosis duration correlates with the glycine conjugation of primary BAs within PD-D subjects alone (*r* = −0.42, *p* = 0.015), whereas PD-D shows only a non-significant difference compared to PD-MCI and PD-CN at the similar levels of diagnosis duration. This conclusion is also supported by the fact that the two measured glycine-conjugated primary BAs (GCA and GCDCA) are associated with the diagnosis duration but not with PD-D, and on the other hand, other BA-related analytes that are significantly different in PD-D are unrelated to glycine-conjugated primary BAs and are not significantly associated with the disease duration.

Motor section score of the Unified Parkinson’s Disease Rating Scale (UPDRS-M)^21^ is associated with decreasing α-aminobutyrate synthesis in both brain regions (Table 2). Unified Staging System for Lewy Body Disorders (USSLB)^22^ score is associated with a marker of carnitine-acylcarnitine translocase deficiency in cortex.

The remaining results are significantly associated only in putamen: USSLB score is associated with decreasing glutamine (increasing glutaminase activity), betaine, lactate, as well as glycine conjugation of chenodeoxycholic acid and sum of neurotransmitters (Table 2). Global senile plaque density shows significant associations with decreasing threonine, glutamine (increasing glutaminase activity and glutaminolysis rate), serotonin, and two lipid species – PC aa C28:1 and TG(22:6_32:1). Mini-Mental State Examination (MMSE) score^23^ is associated with decreasing α-aminobutyrate synthesis and indicator of short/branched-chain acyl-coenzyme A dehydrogenase deficiency, and with increasing betaine and several indicators with phenylalanine.

There was no statistically significant association with neurofibrillary tangles (Table 2). We mentioned several analytes in association with multiple scores, so we investigated these relationships further by testing correlation between the scores and by combining them into a mutual regression model. MMSE and USSLB do not correlate well (*r* = −0.19, *p* = 0.34) and remain highly significant (both *p* = 0.002) in a combined regression model for betaine. Therefore, betaine seems to be associated with both scores independently. MMSE and UPDRS-M correlate very mildly (*r* = 0.28, *p* = 0.20), but only UPDRS-M (*p* < 0.0001), and not MMSE (*p* = 0.11), remains significant in a combined regression model for α-aminobutyrate. Therefore, UPDRS-M is the main score associated with α-aminobutyrate, not MMSE. Accordingly, only the association with UPDRS-M was significant in both brain regions. USSLB and senile plaque density show a medium correlation (*r* = 0.40, *p* = 0.021) and both scores remain significant (*p* = 0.03, 0.007) in a combined regression model for glutamine. The size of the effects also remain similar (*β* = −0.40, −0.51) and we cannot tell whether the significance is due to the correlation or due to independent associations with glutamine. However, observing the trends in cortex, glutamine and related indicators show more consistent effects in association with senile plaque density rather than USSLB.

## Discussion

To the best of our knowledge, this is the largest metabolomic study in PD with human brain tissue to date and the largest metabolomic study in human putamen in general. Our results reveal metabolic changes associated with PD, with specific cognitive subgroups, with levodopa, and with clinical and pathological scores of disease progression. The affected metabolic pathways include neurotransmitters and bile acids in PD; glutamate metabolism, polyamines, and betaine metabolism in PD with cognitive impairment; while differences in one-carbon metabolism, LPCs, cholesteryl esters, ACs, TCA cycle, and β-alanine metabolism are associated with acute levodopa presence. Some levodopa-related changes further exhibit an apparent temporal delay in PD-D: spermidine, FAs, PCs, and GCs. Several microbiome-derived metabolites are also differentially present in brain. Levodopa-related homocysteine accumulation in brain best explains the dementia status in PD.

Reduced serotonin synthesis in PD alongside dopamine reflects the degeneration of both neurotransmitter systems. Serotonergic impairment in PD is well-known but poorly understood^24^. We show that the decrease in serotonin is apparent regardless of cognition status but we found a significant association with senile plaque formation. This is in line with the evidence that stimulation of serotonin receptors leads to decrease in amyloid plaque formation via activation of α-secretase^25^. The reason behind the serotonergic impairment is unclear and it may be a sign of broader neurodegeneration where dopaminergic neurons are the most but not the only impacted neurons. Some authors suggest levodopa contribution, especially since it is processed in both dopaminergic and serotonergic neurons, and several mechanisms of levodopa toxicity on the serotonergic system have been proposed^26^. This process would happen as a result of long-time levodopa exposure and would likely not be reflected in momentary DOPA levels that fluctuate as the medication is absorbed and eliminated from the body. We see a mild, non-significant trend of decreased serotonin with increased DOPA in cortex (*r* = −0.20, *p* = 0.11). Alternatively, we cannot exclude the possibility that a portion of carbidopa is passing through the blood-brain barrier when its integrity is impaired, directly inhibiting serotonin synthesis. Disrupted blood-brain barrier has been associated with PD^27^.

PLA2 plays an important role in regulation of phospholipid metabolism and inflammation. The observed reduction in indicator of PLA2 activity in PD cortex is, however, likely a result of changes in composition of PCs (with elevation of specific diacyl PCs) rather than the absolute PLA2 activity and its production of LPCs and arachidonic acid, as they remain unaltered.

The detection of bile acid changes in brain confirms the existence of gut-brain cross-talk. Primary BAs are produced in liver, secreted into small intestine when needed, and later reabsorbed. Meanwhile, gut bacteria can convert them into rather pro-inflammatory secondary BAs. Conjugation with amino acids readily occurs in liver as well as microbiota^28^, and changes in the BA composition have been associated with various pathologies^29^, including AD in our previous research^30^. The involvement of BAs in PD is also suspected^31^ and has been connected with microbial dysbiosis^32^. Our results show increased glycine conjugation of secondary bile acid GDCA across the cognitive groups, and progressive decline in glycine conjugation of primary BAs with respect to the disease duration (cortex) and USSLB (putamen). This is consistent with reports of elevated GDCA in PD plasma^33,34^ and gut bacteria changes related to reduced primary BA biosynthesis^35^. Worth noting is the connection with coenzyme A (CoA) in the process of BA conjugation^36^, since our pathway analysis detected a disturbance in pantothenate and CoA biosynthesis, at least in the cognitively impaired groups. Inadequate amount of CoA would slow down the rate of primary BA conjugation in liver. Indeed, decreased pantothenate (vitamin B5), the precursor of CoA, has been observed in PD brain^7^.

We found no significant disturbances in PD related to diacylglycerols, glycerophospholipids, and sphingolipids reported by previous studies in cortex^10,11^ although we found changes in glycerophospholipids and sphingolipids in association with acute levodopa presence as discussed further in sections devoted to levodopa.

**Fig. 5:**
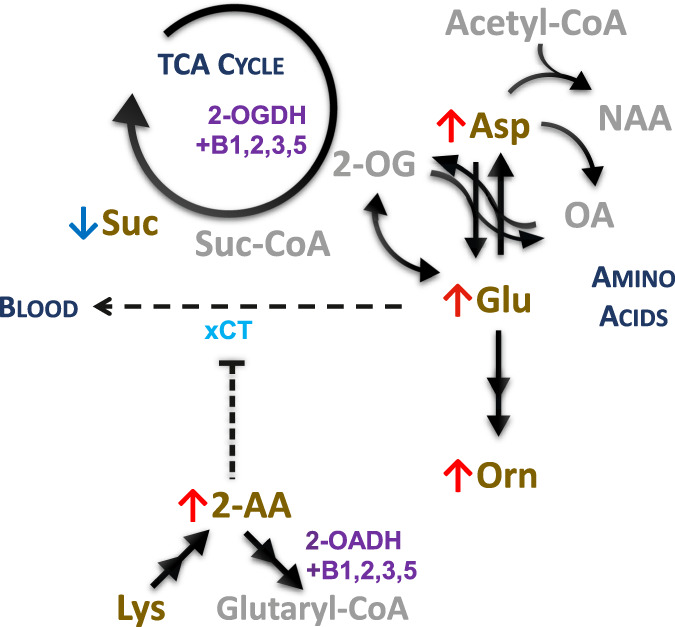
Glutamate metabolism and related pathways altered in PD with cognitive impairment. Selected metabolic reactions pertaining to glutamate metabolism and its connection with α-aminoadipate and observed changes. Arrows: red – upregulated; blue – downregulated; black solid – metabolic reaction; black sparsely dashed – export from brain; black densely dashed – inhibition. Font: gold – measured metabolite; gray – metabolite not included in the assay; purple – enzymes and cofactors; bright blue – transporters. 2-AA α-Aminoadipate, 2-OA 2-Oxoadipate, 2-OG 2-Oxoglutarate, Asp Aspartate, B*n* Active form of vitamin B*n,* CoA Coenzyme A, DH Dehydrogenase complex, Glu Glutamate, Lys Lysine, NAA N-Acetylaspartate, OA Oxaloacetate, Orn Ornithine, Suc Succinic acid, TCA Tricarboxylic acid, xCT Cystine/glutamate antiporter.

There are several interconnected areas of metabolism altered in PD-CI groups, as depicted in Fig. 5. Glutamate and aspartate are mutually convertible and both metabolites are significantly elevated. Glutamate accumulation is neurotoxic and its involvement in PD has long been suspected^37^. Glutamate can be converted into 2-oxoglutarate and consumed in the tricarboxylic acid (TCA) cycle via 2-oxoglutarate dehydrogenase (OGDH) complex. However, our results suggest a bottleneck in the TCA cycle as evident by the decreased succinate. OGDH complex function depends on active forms of several B vitamins, including CoA, and its dysfunction would compromise the cellular energy metabolism and homeostasis. Glutamate and aspartate do not pass the blood-brain barrier into the brain, but glutamate passes outside through cystine/glutamate antiporter^38^, an important system in the clearance of excessive glutamate and import of cystine, a precursor of antioxidant glutathione. The cystine/glutamate antiporter system is inhibited by α-aminoadipate^39^, which we found elevated, thus potentially impairing the antiporter function. A particularly sensitive area for its dysfunction is retina^40^, and consistent with our hypothesis, retinal damage has been recently proposed as an early marker of PD^41^. Additionally, changes in DNA methylation of the antiporter gene SLC7A11 have been associated with a risk of PD^42^ and lower glutathione levels have been reported in PD brain^6^. The reason for increased α-aminoadipate is not obvious. Interestingly, its degradation pathway employs 2-oxoadipate dehydrogenase complex (OADH), which is very similar to OGDH complex including shared subunits^43^ and dependence on B vitamins and CoA. We hypothesize that the observed changes are caused by dysregulation in these complexes, possibly due to low levels of vitamin B5 and CoA. Accordingly, N-acetylaspartate is acetyl-CoA-dependent product of aspartate/glutamate metabolism and has been found downregulated in imaging studies^44^, implying a bottleneck, whereas ornithine is a CoA-independent product of glutamate. Interestingly, we found the indicator of ornithine synthesis increased similarly as glutamate, showing undisturbed propagation of the glutamate change.

Ornithine is further metabolized into putrescine through the action of the rate-limiting enzyme ornithine decarboxylase, which was decreased in PD-CI putamen. A previous investigation of basal ganglia found no differences in putrescine in PD^9^ but the number of samples was small, and cohorts differed in post-mortem collection intervals. Putrescine has been detected and shown to be decreased in red blood cells in PD^45^ but elevated in cerebrospinal fluid (CSF)^46,47^, although these results might be confounded by age. Another metabolomic study in serum found no differences in putrescine in PD^48^. There is also a report of putrescine as one of the metabolites contributing to diagnosis of dementia status in PD^49^. Since the change that we observed is not propagated to other polyamines, it might not have any important biological effect. However, it could be a surrogate marker of a deficiency in pyridoxal 5-phosphate (PLP), the active form of vitamin B6, which acts as a cofactor for ornithine carboxylase. We observed other signs of potentially low PLP in brain of subjects with cognitive impairment in our study of one-carbon metabolism^14^. Lower plasma B6 has been previously associated with PD as well^50^.

Betaine is also related to one-carbon metabolism, as it facilitates re-methylation of the toxic sulfur amino acid Hcy, although its functions are wider and exhibit anti-inflammatory, anti-apoptotic, and anti-diabetic properties^51^. We previously reported decreased concentration of betaine in cortex of cognitively impaired subjects, both PD and AD^14^. Here, we confirm that the same reduction exists in PD putamen and is independently associated with cognitive decline (MMSE) and spread of histopathological changes (USSLB). Given the interesting properties of betaine, it is plausible that its deficiency directly contributes to neurodegeneration. Indeed, there are reports showing benefits of betaine supplementation on cognition^52,53^.

There are several altered metabolic indicators related to phenylalanine, with the largest difference in lower tyrosine/phenylalanine ratio, mainly due to higher phenylalanine. This indicates an impaired function of phenylalanine hydroxylase, a crucial enzyme for dopamine synthesis. Its dysfunction in PD is a known phenomenon and is considered a secondary event reflecting the death of dopaminergic neurons^54^. Similarly, we found several metabolic indicators with phenylalanine associated with MMSE score, with cognitive decline always in the direction of higher phenylalanine concentrations.

Some changes associated with scores of disease progression have already been mentioned. Another strong relation is between UPDRS-M motor symptoms progression and lower α-aminobutyrate. This metabolite is a downstream product of catabolism of threonine and cystathionine and has been directly related to glutathione compensation against oxidative stress^55^. In PD, lower levels of α-aminobutyrate have been previously observed in cerebrospinal fluid^56^. Given the decreased levels of glutathione in PD brain^6^, it is possible that our finding of the association with α-aminobutyrate reflects the protective effect of glutathione on deterioration of motor control in PD.

Senile plaque density is associated with decreasing glutamine. This observation is consistent with the literature, where glutamine synthetase has been found downregulated in AD astrocytes especially near the senile plaques and without the reduction of astrocyte count^57^. Dysregulated glutamine metabolism promotes glutamate toxicity and neurodegeneration, which can occur in response to inflammatory cytokines^58^. The association of USSLB with decreasing lactate is also consistent with astrocytic impairment. Lactate, in brain produced mainly by astrocytes, is an important energy source for neurons. Astrocytes mediate α-synuclein clearance, and this mechanism is impaired in a known genetic form of PD^59^. It has been shown that lactate helps activate autophagy and rescue cells in a PD model^60^.

There are several more associations with senile plaque. Triglyceride TG(22:6_32:1) and phosphatidylcholine PC aa C28:1 decrease with higher plaque load in PD putamen. One chain of TG(22:6_32:1) is formed by ω-3 docosahexaenoic acid (DHA), which is thought to impede amyloid production^61^, so inadequate levels of DHA trafficked into brain could accelerate plaque formation. The meaning of the association with PC aa C28:1 is unclear. Similarly for threonine although the pattern is a bit different: the PD subjects with lower plaque density in putamen have levels in the higher range of controls, whereas those with higher plaque density have threonine in the lower range of controls. This relationship is interesting, raising a question whether threonine can have a protective effect on senile plaque formation in PD or if it mirrors another underlying mechanism.

Increasing marker of short/branched-chain acyl-CoA dehydrogenase deficiency with decreasing MMSE score suggests affected mitochondrial β-oxidation via downregulation of 2-methylbutyryl-CoA dehydrogenase. Its dysfunction promotes lipid oxidative damage and depletes glutathione levels^62^. Thus, it is possible that downregulation of this enzyme contributes to cognitive impairment, especially in the presence of concomitant insults as in PD. Fatty acid β-oxidation impairment is further indicated in cortex in association with higher USSLB score, where the marker of carnitine-acylcarnitine translocase deficiency suggests a lower activity of this enzyme as the pathology spreads.

The effect of levodopa medication on metabolism is not well explored and there is a debate whether its side effects accelerate neurodegeneration^63^. There are reports of affected glucose and fatty acid (FA) metabolism^64,65^, lipid peroxidation^66^, as well as increased stress on proteolytic^67^ and lysosomal^68^ systems due to DOPA incorporation into proteins, resulting in mitochondrial dysfunction. Levodopa and carbidopa also sequester PLP, an important cofactor for many biological processes, and have been shown to reduce plasma PLP in PD^69^. We have previously described levodopa-induced changes in methylation and τ-protein phosphorylation in mice^19^ and showed levodopa-related Hcy accumulation in brain cortex of human PD subjects as characteristic of their dementia status^14^.

The current study found no better discriminator of dementia in PD than the levodopa-related Hcy accumulation, supporting the importance of its direct link to the dementia in PD. Plasma Hcy can be lowered by supplementation of certain B vitamins^70^ and the same effect is also expected in brain (due to the presence of all vitamin B-dependent Hcy-related pathways), which is supported by high brain Hcy levels in a mouse model for B12 deficiency^71^. Several other changes were related to methylation (arginine methylation, betaine, and sarcosine), which might be explained by disproportion in S-adenosylhomocysteine (SAH) and S-adenosylmethionine caused by levodopa methylation.

LPCs, together with arachidonic acid, are produced by PLA2, the activity of which is increased during inflammation^72^, and have a potentially detrimental effect on mitochondria^73^. The increase of LPCs in PD L+ putamen, along with a similar although non-significant trend in arachidonic acid, suggests a possible levodopa-related inflammatory process. LPCs have been previously associated with upregulated cholesterol biosynthesis^74^, consistent with our finding of increased MUFA-CEs in L+ putamen.

The observed decrease in LCFA-ACs in L+ putamen reflects a disturbance in FA β-oxidation, whether due to diminished substrate availability (CoA, FAs) or downregulated function of FA transport into mitochondria or peroxisomes. Similar LCFA-ACs decrease has been observed in plasma of PD subjects as compared to controls^75^ although not directly correlated with levodopa. Here, we showed clear levodopa-related downregulation in putamen, with a similar but non-significant trend in cortex. Any energy metabolism disruption can negatively impact cell survival during cellular stress as suspected with levodopa.

Surprising is the finding of apparent phase delay in the levodopa cyclical effect in cortex of PD-D subjects, potentially starting to appear already in PD-MCI. Since our L+/L− classification is based directly on DOPA levels in the tissues, this effect cannot be caused by delayed drug absorption or transport into brain. The differential response in one-carbon metabolism with Hcy (and SAH) accumulation in PD-D might play a role, as there are multiple regulatory mechanisms inhibiting numerous methyltransferases, including DNA methyltransferases affecting gene expression^76^, and increasing Hcy clearance. Therefore, we hypothesize that the levodopa effect can show variable delay depending on the magnitude of Hcy accumulation, strength of the initiated regulatory response, and speed of Hcy clearance – parameters which are different between the PD cognitive groups. This observed effect warrants further investigation. Temporal phenomena can be ideally confirmed in a longitudinal study. However, this might not be realizable with human brain tissue (using a similarly invasive technique) and less powerful approaches might be necessary, including larger association studies or investigations with animal models.

Nevertheless, we detected this pattern across multiple classes of metabolites. The changes in fatty acid are mainly due to decreased MUFAs. This would be consistent with reduced lipolysis, a consequence of Hcy metabolism generating cysteine^77^, and a possible cause of disturbed β-oxidation. Decreased ratio of acyl-alkyl/diacyl PCs then points towards reduced function of alkylglycerone phosphate synthase or fatty acid reductase. These enzymes are located in peroxisomes, so the change might reflect peroxisomal dysregulation. Hcy might be the cause via interference with peroxisome proliferator-activated receptor methylation^78^. Several decreased ceramides and sphingomyelins were previously found in PD putamen^12,13^ and we see their decrease in relation to levodopa. Altered ratios of ceramides, GCs, and SMs suggest impeded degradation of 3GCs to simpler GCs, which are directed by lysosomal enzymes α-galactosidase and β-glucocerebrosidase. Lysosomal clearance can also be reduced by homocysteine^79^, and moreover, lysosomal dysfunction further attenuates peroxisomal function^80^. Furthermore, decreased spermidine can downregulate lysosomal and autophagosomal function^81^ as well as mitochondrial respiration^82^. Spermidine is connected to the methionine cycle through PLP-dependent SAM decarboxylase and it is unclear whether the levodopa-carbidopa medication can directly interfere with this pathway through PLP sequestration or changes in SAM. Lower levels of spermidine have been previously reported in CSF of PD subjects^47^.

The oscillation of β-alanine metabolism results in notably lowered anserine (and to a similar degree but without statistical significance carnosine) levels in PD-CI cortex. These histidine dipeptides are important antioxidants and have been previously associated with cognitive status^83^ and PLP availability^84^. Interestingly, the changes in PD-D include muscle-related τ-methylhistidine and might be an indicator of faster muscle catabolism, while in PD-ND, diet-related π-methylhistidine^85^ is more affected.

Finally, the group of microbiome-related metabolites consisting of para-cresol sulfate, indoxyl sulfate, and trimethylamine N-oxide fluctuates in PD-CI, especially dementia. These compounds are considered metabotoxins and have been previously found elevated in PD biofluids^86^. We have reported their increase in AD subjects^30^, which strengthens the association of these compounds with cognitive impairment in general. The reason for their interaction with levodopa is not clear and it is conceivable that the medication stimulates growth of DOPA-metabolizing bacteria.

This study has multiple strengths: We analyzed changes directly in brain tissue, which can reveal more information about the neurodegeneration than surrogate measurements in biofluids. The samples had exceptionally low post-mortem collection intervals and were homogeneous across the groups. This is highly important for reliable comparison, as post-mortem degradation processes affect metabolism and energy balance. We were able to analyze PD subjects in relation to cognitive impairment, which revealed interesting changes. Similarly, we investigated the impact of acute PD medication presence, which turned out to have a profound effect on brain metabolism. Furthermore, our analysis considered multiple confounding effects, including diseases notoriously impacting metabolism.

There are also several limitations. By design, this is an association study, which prohibits us from validating causal relationships although we discuss plausible connections with the etiology of PD or related cognitive impairment. For PD-D subjects, we had no putamen tissue, which would help us confirm the consistency of our findings. Not all disease progression scores were known for all subjects and even though we attempted to detect confounding through comparison of associations between PD groups and the progression scores, a larger study would be needed to fully disentangle their individual contributions. Despite analyzing the levodopa impact, this does not capture any potential effect of carbidopa for its substantially larger elimination halftime. No genotyping was performed to identify subjects with a known genetic cause of PD. Next, we did not have any information regarding diet. PD patients are often advised to avoid protein intake immediately with levodopa (due to absorption), which could affect several areas of metabolism. We found some levodopa-related oscillation in π-methylhistidine, a suggested marker for meat consumption^85^, but it was in the opposite direction and did not correlate well with other findings, which would remain significant after adjustment for π-methylhistidine levels. Furthermore, all subjects were non-Hispanic (where ethnicity was provided) White Americans and it is not certain whether the metabolic changes generalize outside this context.

In conclusion, we performed a large metabolomic analysis in human brain. Most of the discovered changes are associated with cognitive impairment, especially glutamate, aspartate, and α-aminoadipate metabolism and one-carbon metabolism with levodopa-related homocysteine accumulation in brain best explaining the dementia status in PD (AUC = 89%, CI_95_ = 75–100%). These alterations may indicate insufficient levels of vitamins B5 (pantothenic acid), consistently with a previous finding^7^, and B6 (pyridoxal 5’-phosphate). Therefore, it is plausible that normalization of B vitamins and homocysteine metabolism in brain would significantly reduce the risk of dementia in PD, which warrants further research. We have also found multiple levodopa-related associations, mainly in lipids, and many of the changes exhibit an apparent temporal delay in PD-D. This interaction and its nature should be further studied and validated since its omission may mask the levodopa effects if confirmed.

## Methods

This is a targeted metabolomic case-control study in PD with post-mortem human brain tissue using liquid chromatography and mass spectrometry.

### Study subjects

From the Banner Sun Health Research Institute brain bank (Sun City, AZ, USA)^87^, we obtained post-mortem brain frontal cortex samples (Brodmann area 9; 500 mg) of 36 cognitively normal controls and 65 PD subjects at various stages of cognitive impairment: 32 with dementia (PD-D) and 33 without dementia (PD-ND) subdivided into 19 with MCI (PD-MCI) and 14 cognitively normal (PD-CN). We also obtained post-mortem putamen samples (20 mg) of the identical 35 cognitively normal controls (1 sample was destroyed during analysis) and 33 PD-ND subjects. The numbers were based on available tissue in the biobank with required characteristics and post-mortem collection interval range, combined with allocated funding, and exceed the size of most studies with human brain tissue. The samples were collected between 2004 and 2018 from deceased donors, flash frozen with post-mortem collection interval averaging 3 h, and continuously stored at −80 °C. The diagnosis followed clinical records and histopathological examination: PD subjects had two of the three cardinal clinical signs of resting tremor, muscular rigidity, and bradykinesia, along with pigmented neuron loss and presence of Lewy bodies in substantia nigra, and were treated with levodopa-carbidopa medication. The status of dementia and MCI corresponds to clinical diagnosis using a global Clinical Dementia Rating scale^88^, defining MCI as score 0.5 and dementia as score 1 or higher. To differentiate from Alzheimer’s type of dementia, the subjects scored no more than “low likelihood of AD” in NIA-Reagan classification^89^. Controls were without a history of cognitive impairment and parkinsonism. All subjects were White Americans, either non-Hispanic or with ethnicity not provided. No other major pathologies of the central nervous system were present.

Clinical profiles with assessment of progression scores were updated during the last pre-mortem visit. Histopathological density scores of neurofibrillary tangles and senile plaque (neuritic, cored, and diffuse) were evaluated using templates of the Consortium to Establish a Registry for Alzheimer’s Disease (CERAD)^90^ and the scores were summed over five brain regions: frontal, temporal, parietal, entorhinal, and hippocampal CA1 region.

Basic sociodemographic and clinical characteristics are summarized in Table 3. Sociodemographic variables show either no differences between the groups or minor differences with a good overlap. Levodopa medication dosage is similar across the PD groups although this information is available only for a third of the subjects. Scores of disease progression and histopathological examination show major differences as expected. Senile plaque seems to be elevated only in PD-ND groups. UPDRS-M score and PD duration are particularly higher in PD-D group, which is a concern for a possible confounding effect. As the UPDRS-M score is known only for 57% of PD subjects, we could not include the score in the main differential regression for PD groups. Instead, as described in the Statistical analysis section below, we analyzed disease progression scores separately and then compared the results to detect possibly confounded associations.

**Table 3:** Sociodemographic and clinical characteristics of the cohort.

	Groups				*P*-value	
	Controls (*N* = 36)	PD-CN (*N* = 14)	PD-MCI (*N* = 19)	PD-D (*N* = 32)	All groups	PD groups
PMCI, hours, mean (SD)	3.2 (1.0)	3.3 (0.9)	3.4 (1.2)	3.4 (0.9)	0.78	0.85
Freezer storage, years, mean (SD)	10 (4)	8 (4)	6 (4)	8 (4)	0.005	0.31
Sex						
Male, No. (%)	22 (61)	8 (57)	14 (74)	24 (75)	0.49	0.52
Female, No. (%)	14 (39)	6 (43)	5 (26)	8 (25)		
Ethnicity						
Non-Hispanic, No. (%)	32 (89)	14 (100)	16 (84)	28 (88)	0.59	0.44
Not provided, No. (%)	4 (11)	0 (0)	3 (16)	4 (13)		
Race	All White				1	1
Age, years, mean (SD)	82 (10)	85 (6)	82 (6)	80 (5)	0.057	0.033
Education, years, mean (SD)	14 (3)	16 (3)	14 (3)	16 (3)	0.039	0.073
BMI, kg/m^2^, mean (SD)	25 (5)	22 (4)	25 (8)	27 (9)	0.042	0.024
APOE, ε4 allele, No. (%)	5 (14)	2 (14)	3 (16)	3 (9)	0.86	0.70
PD duration, years, mean (SD)	–	11 (7)	12 (6)	18 (9)	–	0.008
Dementia onset, years, mean (SD)	–	–	–	75 (6)	–	–
Levodopa dosage, mg/day, mean (SD)	–	450 (217)	500 (173)	509 (226)	–	0.86
UPDRS-M score, mean (SD)	7 (5)	32 (15)	31 (15)	50 (16)	<0.001	0.005
USSLB score, mean (SD)	0.0 (0.0)	3.0 (0.5)	3.7 (0.5)	3.4 (0.6)	<0.001	0.002
MMSE score, mean (SD)	28 (1)	28 (1)	25 (3)	20 (6)	<0.001	<0.001
Neurofibrillary tangle density, mean (SD)	2.9 (1.6)	5.3 (2.6)	5.2 (1.6)	4.8 (2.1)	<0.001	0.68
Senile plaque density, mean (SD)	2.7 (3.9)	4.6 (4.7)	5.1 (6.0)	1.8 (3.3)	0.056	0.025
Hyperlipidemia, No. (%)	13 (36)	7 (50)	9 (47)	11 (34)	0.65	0.49
Diabetes mellitus, No. (%)	12 (33)	1 (7)	3 (16)	8 (25)	0.21	0.34
Renal insufficiency, No. (%)	5 (14)	3 (21)	2 (11)	5 (16)	0.87	0.68
Hypothyroidism, No. (%)	9 (25)	3 (21)	5 (26)	5 (16)	0.74	0.61

Levodopa dosage is unambiguously known only for 31% of PD subjects. MMSE score is known for 88% of PD subjects and 75% of controls. UPDRS-M score, measured in off-levodopa state, is known for 57% of PD subjects and 67% of controls. P-values are listed for comparison between all groups and between PD groups only.

*APOE* Apolipoprotein E, *BMI* Body-mass index, *CN* Cognitively normal, *D* Dementia (non-Alzheimer’s type), *MCI* Mild cognitive impairment, *MMSE* Mini-Mental State Examination, *PD* Parkinson’s disease, *PMCI* Post-mortem collection interval, *UPDRS-M* Motor section of the Unified Parkinson’s Disease Rating Scale, *USSLB* Unified Staging System for Lewy Body Disorders.

There are no differences in several disorders that impact metabolism neither in the frequency of apolipoprotein E ε4 allele, which is consistent with the histological confirmation that cognitive impairment of the PD subjects is unrelated to AD (where the allele frequency is several times higher^91^). Importantly, post-mortem collection intervals are very homogeneous between the groups. Freezer storage since collection is somewhat higher for the control samples, but we did not observe any signs of related tissue degradation (e.g. choline levels of the longer stored control samples were indistinguishable from those with shorter storage; two-tailed Welch’s t-test *p*-value = 0.72). Besides, we controlled for any freezer storage effect in the regression alongside other covariates.

### Ethics statement

The authors purchased all tissue samples from the Banner Sun Health Research Institute brain bank (Sun City, AZ, USA), which manages tissue collection and deposition, records written informed consents from all subjects prior to their death, and pledges to perform all methods in accordance with relevant guidelines and regulations under approval by the Banner Sun Health Institutional Review Board^87^. The authors obtained prior approval from the Banner Sun Health Research Institute brain bank to use the tissue samples for research purposes and adhered to relevant guidelines and regulations.

### Chromatography and mass spectrometry

Targeted metabolomic analysis was based on triple quadrupole ultra-high-performance liquid chromatography tandem mass spectrometry (UHPLC-MS/MS) using Shimadzu Nexera chromatography platform (Shimadzu Corporation, Kyoto, Japan) coupled to Sciex QTrap 5500 mass spectrometer (AB Sciex LLC, Framingham, Massachusetts, USA). We applied the Biocrates MxP Quant 500 targeted kit (Biocrates Life Sciences AG, Innsbruck, Austria), potentially quantitating 106 small molecules and free fatty acids in chromatography mode and 524 complex lipids in positive flow-injection mode (FIA-MS/MS), exploring a broad range of metabolic pathways. Annotations for the individual metabolites with identifiers to external databases are provided in Supplementary File 3. However, Biocrates prefers to keep mass transitions of individual metabolites undisclosed as proprietary knowledge.

Additionally, 232 metabolic indicators were calculated from sums or ratios of relevant metabolites according to Biocrates MetaboINDICATOR formulas^92^ (see Supplementary File 3). We refer to the whole set of metabolites and metabolic indicators as analytes. The indicators can be regarded as physiologically relevant measures and are statistically analyzed separately from metabolites. The indicators denoted as “X synthesis” are computed as a ratio of metabolite X and its main precursors in an attempt to reflect the conversion ratio. Since there are multiple explanations why such an indicator could be altered, the interpretation needs to be done cautiously in context of the individual metabolites.

Brain cortex samples were extracted in plastic vials with 85% ethanol in phosphate-buffered saline at concentration 3 µl/mg, homogenized with sonicator, and centrifuged for 20 minutes. The clear extract (2 × 15 µl for cortex, 15 µl for putamen) was transferred onto a kit plate with pre-injected internal standards and dried down. In brief, the rest of the assay includes derivatization with 5% phenylisothiocyanate in pyridine, ethanol and water (1:1:1), and subsequent extraction with 5 mM ammonium acetate in methanol. Chromatography was done with 0.2% formic acid in acetonitrile (organic mobile phase) and 0.2% formic acid in water (inorganic mobile phase). Flow-injection analysis was performed with methanol and Biocrates MxP Quant 500 additive of undisclosed composition. All solvents used were of LC/UHPLC-MS grade, except for ethanol with the American Chemical Society and United States Pharmacopeia grade.

Sample handling was done on dry ice to avoid multiple freeze-thaw cycles. We randomized the samples across plates, with stratification, already prior to their processing to avoid any accidental bias towards one of the subject groups. Plates included blanks to calculate limits of detection, repeats of a kit quality control sample to calculate concentrations and monitor the coefficient of variation (median for analyzed compounds: <10% for UHPLC, <20% for FIA), and kit calibrators for seven-point calibrations of certain compounds.

### Data preprocessing

#### Peak areas and concentrations

Mass spectrometry signal was acquired in Sciex Analyst v1.6.24 (AB Sciex LLC, Framingham, Massachusetts, USA) and chromatographic peaks were identified and integrated in Biocrates MetIDQ Oxygen-DB110-3005 (Biocrates Life Sciences AG, Innsbruck, Austria). We have reviewed the integration process using the same software by checking integration boundaries for captured signal of every metabolite and internal standard across the samples to confirm that whole peaks were captured and that shared peaks were integrated consistently. Areas of metabolite peaks were divided by areas of their respective internal standards (Supplementary File 3). Further processing was done in R v3.6.1^93^ with RStudio v1.2.5033^94^. For most compounds, concentrations were estimated linearly from expected concentrations in the quality control sample using their median. Seven-point quadratic calibration was applied where possible.

#### Plate normalization

Cortex samples were run in two plates and to account for potential batch effects, plates were normalized (per metabolite) by scaling through median normalization: For a given metabolite, values of reference samples in each plate are scaled by such a factor so that their median is equivalent to the median of values of all reference samples before normalization. For this purpose, we were able to leverage information from quality control samples as well as all human samples owing to a simple trick: A copy of values in each subject group and QCs were first divided by the respective group median, upon which all of these pre-normalized samples were used as the reference samples for estimation of the normalization factors. The formal definition of this new extension of median normalization is provided in Eqs. (1) to (3).

1 $$\forall m,s{\rm{;}}\,m\in M,s\in S:\,{r}_{s}^{(m)}={x}_{s}^{(m)}/\mathop{{\rm{median}}}\limits_{t\in {S}^{({g}_{s})}}\left({x}_{t}^{(m)}\right)$$

2 $$\forall m,p{\rm{;}}\,m\in M,p\in P:\,{q}_{p}^{(m)}=\mathop{{\rm{median}}}\limits_{t\in {S}_{p}}\left({r}_{t}^{(m)}\right)/\mathop{{\rm{median}}}\limits_{t\in S}\left({r}_{t}^{(m)}\right)$$

3 $$\forall m,s{\rm{;}}\,m\in M,s\in S:\,{y}_{s}^{\left(m\right)}={x}_{s}^{\left(m\right)}/{q}_{{p}_{s}}^{(m)}$$

Where: $M$ – set of all metabolites

*P* – set of all plates

*S* – set of all samples (subject samples and QCs)

${S}_{p}^{(g)}$ – all samples from plate $p$ and group $g$

*g*_*s*_ – group of sample $s$ (one of the subject groups or QC)

*p*_*s*_ – plate of sample $s$

*q*_*p*_ – normalization quotient for plate $p$

${x}_{s}^{(m)}$ – original value of metabolite $m$ for sample $s$

${r}_{s}^{(m)}$ – pre-normalized reference value for metabolite $m$ and sample $s$

${y}_{s}^{(m)}$ – normalized value of metabolite $m$ for sample $s$

#### Limits of detection (LODs)

LODs were calculated as 2× median signal in blanks. Metabolites with more than 50% values below LOD in all subject groups were filtered out. Values below LOD were not adjusted, since they represent the best estimate of the true values. However, completely zero values were interpolated as half of the minimal non-zero value for a given metabolite to avoid strict zeros, since zero values are more difficultly handled by subsequent transformations as well as biologically unlikely.

#### Calculated analytes

Metabolic indicators were calculated according to Biocrates MetaboINDICATOR formulas^92^. Ratios with zeros were treated as missing values and not included in the analysis. The metabolic indicators were also plate-normalized.

#### Data transformation

In R environment^93,94^, we applied Box-Cox transformation with R package *car*^95^ to better approximate Gaussian distributions. Tukey’s fencing^96^ was used to adjust remote outliers (*k* = 3) to protect against skewing the means by extreme values while not reducing the variance greatly compared to outlier removal. Finally, the values were standardized with respect to control samples to facilitate comparison of regression coefficients in the statistical analysis.

#### Missing values

The statistical analysis requires all regressors to be non-missing. Therefore, several missing sociodemographic values were imputed: Missing body mass index (BMI) values of 4 subjects and length of education of 4 subjects were imputed as a mean value conditional on the subject group and sex. The imputed values were distributed across the groups and sex as follows: BMI – 1× controls male (5%), 1× controls female (7%), and 2× PD-MCI male (14%); education – 2× controls male (9%), 1× controls female (7%), and 1× PD-MCI male (7%). The imputation did not substantially affect the results compared to completely removing the samples, but it achieves higher statistical power.

### Statistical analysis

#### Sociodemographic and clinical characteristics

Key characteristics of subjects and samples in each group were compared with two-tailed Fisher’s exact test for binomial variables and analysis of variance (ANOVA) on a linear model constructed with the R package *nlme*^97^ for continuous variables, not assuming equivalence of variance among the subject groups.

#### Differential analysis

The analysis of differences in analytes between PD groups and controls and the effect of levodopa medication was based on a series of multivariable linear regression models with R package *nlme*^97^, one for each analyte, with its values as dependent variables and subject groups – either separately by cognitive status, or all PD together – followed by the indicator of acute levodopa presence in PD-ND and in PD-D (cortex only) as independent variables, without the assumption of equivalence of variance among the groups. The models further included covariates for age, sex, education, BMI, diagnosis of several frequent disorders affecting metabolism: hyperlipidemia, diabetes mellitus, renal insufficiency, and hypothyroidism, as well as post-mortem collection interval and the total length of freezer storage. The last two covariates were log-transformed to be able to capture any time-related exponential decay. Due to standardization, the regression coefficients have a unit of 1 standard deviation of the distribution of controls.

#### Acute levodopa presence

We define the acute levodopa medication presence (L+) as elevated DOPA levels in PD subjects, which happens due to levodopa medication, and absence (L−) as physiological DOPA levels, which may also be partially contributed by the medication but within the physiological range. The physiological threshold was estimated from normalized DOPA concentrations as 95% quantile of the values of controls in the respective brain region. We have previously established that the effect of acute levodopa medication presence differs in demented and in non-demented subjects in this cohort^14^, so we included this interaction in the differential analysis as two regressors – Boolean indicators of acute levodopa presence in PD-D subjects and separately in PD-ND subjects. Given that all subjects were chronically taking the medication, we expected that the acute levodopa presence at the time of death is a result of a random process and not directly related to disease progression scores, thus resulting in no differences in the scores between L+ and L− PD-D/ND subjects. We have verified this assumption using two-tailed Welch’s t-test and found no significant differences except for a potential difference in senile plaque score in the PD-D group (L+ vs L− *p* = 0.04) although observing such a difference is expected due to multiple hypotheses testing (FDR = 0.50) and is consistent with the random process assumption. There were no significant associations with senile plaque score in cortex, so no confounding effect is suspected.

#### Progression analysis

Associations between scores of disease progression and cognitive decline among PD subjects followed a procedure similar to the differential analysis, with the independent variable being a progression score instead of subject groups and levodopa presence. The regression models were constructed so that the controls contributed to the estimation of the effect of covariates but not the progression score. Regression coefficients are two-way standardized: 1 unit corresponds to the change in the value of the analyte by 1 SD of the values of controls with the progression score being changed by 1 SD of values of PD subjects.

#### False discovery rate (FDR) control

For each regression coefficient of interest, two-tailed p-values across the models were controlled for FDR using the q-value procedure with R package *qvalue*^98^. The control was done separately for each combination of plate analytical method (UHPLC/FIA) and analyte type (measured/calculated). Effects with FDR ≤ 0.05 were considered statistically significant.

#### Collinearity check

Assessment of collinearity among all regressors was based on the magnitude of Pearson’s correlation coefficients and adjusted generalized variable inflation factor (GVIF) calculated with the R package *car*^95^. We found no evidence of significant collinearity (all Pearson’s |r | < 0.6 and adjusted GVIFs < 1.5).

#### Pathway analysis

We downloaded definitions of human metabolic pathways from KEGG database^20^ as publicly available on December 07, 2021 and matched them with the measured metabolites. Since certain measurements in the performed assay may represent multiple isoforms undistinguishable by the mass spectra and each isoform can have its own annotations and pathway memberships, we accounted for this by assigning the measured metabolites into all pathways with any of the possible isoforms of the metabolite. Several metabolites remained unassigned to any pathway, especially the ones related to microbial activity. Therefore, we created a custom metabolite set with only microbial metabolites (indoles, 5-aminovaleric acid, trimethylamine N-oxide, para-cresol sulfate, and secondary bile acids). Only metabolic pathways with 4 or more assigned metabolites were considered. We followed the statistical approach of ChemRICH set enrichment analysis^99^, which relies on application of one-sided Kolmogorov-Smirnov test over the distribution of p-values of metabolites assigned to the same set (pathway) using the uniform distribution as a reference. The advantage of this approach is that the test is done over p-values, which can be obtained from any comparative model, in our case the main regression model, so the covariates are considered. This is in contrast with currently available pathway tools, which, besides having problems with pairing multiple isoforms to a single measurement, cannot include covariates in the analysis, resulting in less effective analysis or potentially false positive results. We also performed FDR control via q-values^98^.

#### AUC analysis

We performed a simple univariate AUC analysis using R package *pROC*^100^ to distinguish between PD subjects with and without dementia, with and without interaction with acute levodopa presence. The best score is reported. Confidence intervals are computed using the default DeLong method.

### Reporting summary

Further information on research design is available in the Nature Research Reporting Summary linked to this article.

## Supplementary information

Supplementary Figures

Reporting Summary

Supplementary Data Files

## Data Availability

The authors declare that the data supporting the findings of this study are available within the manuscript: Measured area ratios along with calculated concentrations and metabolic indicators as well as sociodemographic and clinical information are provided in the Supplementary File 4.

## Code availability

Programming code and scripts used for data processing and statistical analysis are available upon request for the purpose of replication of the study results and can be further used or shared only after obtaining an approval from the corresponding author.
