# Supplementary material for: Targeted metabolomic analysis in Parkinson’s disease brain frontal cortex and putamen with relation to cognitive impairment
Source: NPJ Parkinsons Dis. 2023 Jun 3;9:84. doi: 10.1038/s41531-023-00531-y (PMC10239505; doi:10.1038/s41531-023-00531-y)
Supplement: Supplementary file 1 — Supplementary Figures [file 41531_2023_531_MOESM1_ESM.pdf]

Supplementary Figures

Supplementary Fig. 1: Effect of acute levodopa medication presence on metabolism in PD groups in FIA metabolites.

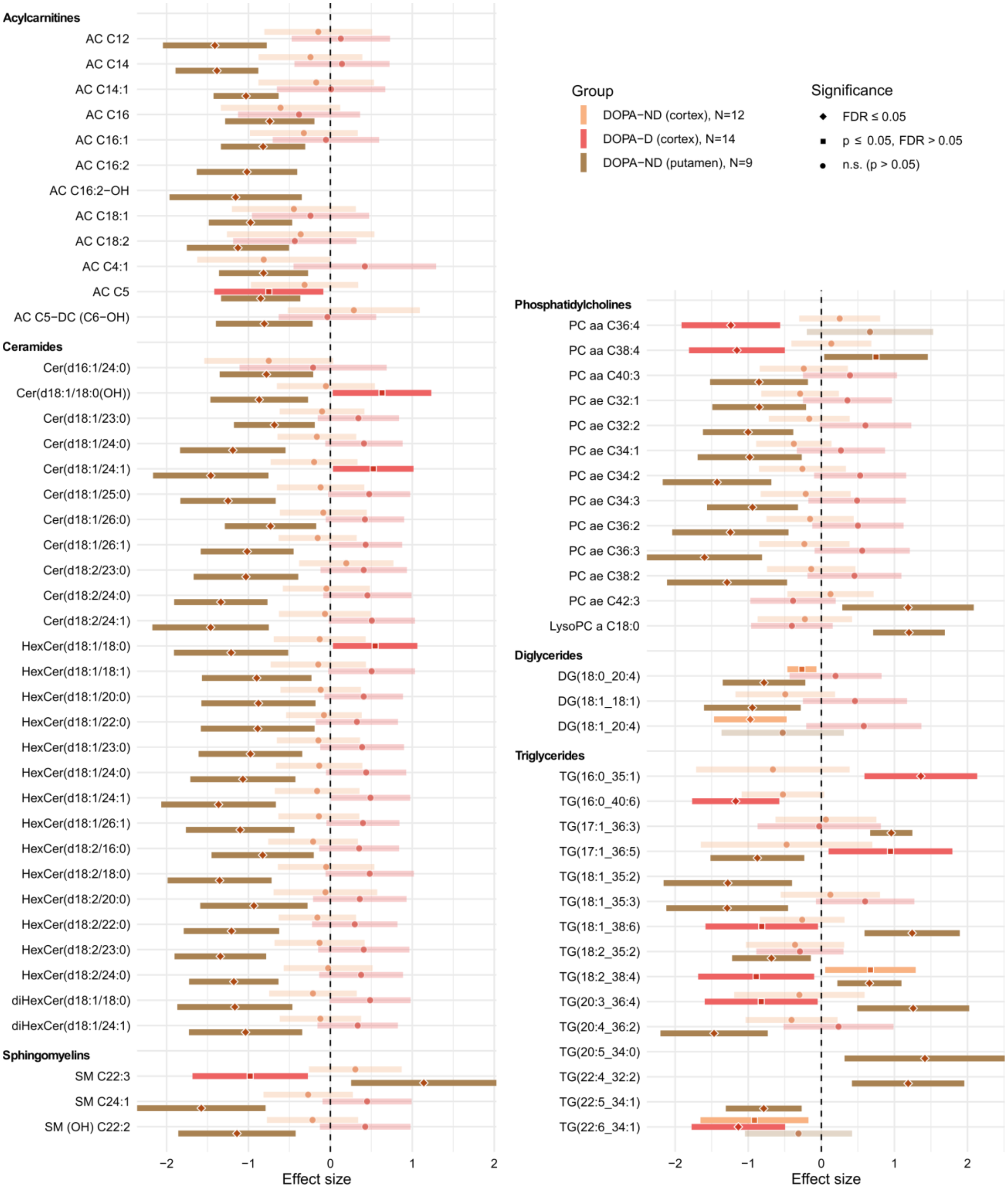

Forest plot for differentially detected metabolites in the FIA part with regression coefficients for the acute levodopa presence in PD in interaction with dementia status (ND – no dementia; D – with dementia) and brain areas. These effects are additive to the PD group effects (Fig. 1 and 2) for subjects with acute levodopa presence, as the regression parameters were a part of the same linear regression

models. The “N” in the legend denotes the number of samples effectively used to estimate each regression parameter. Values are normalized regression coefficients (depicted as the central points; the shape reflects the significance) with 95% confidence intervals (horizontal range lines; lower opacity for non-significant coefficients). The dashed vertical black line represents a zero effect, i.e. equivalent to PD subjects with physiological DOPA levels (in a given group). Every analyte listed is statistically significant (FDR ≤ 0.05) for levodopa medication in putamen or cortex in interaction with dementia status. Pathways are grouped by similarity in the levodopa effect: (a) in all samples or putamen only, (b) in PD with dementia cortex or similarly in putamen, and (c) in PD with dementia cortex and differently in putamen (upon interpretation with Fig. 1 and 2). The regression models included covariates as detailed in the Methods.

Supplementary Fig. 2: Levodopa-associated homocysteine elevation in PD-D.

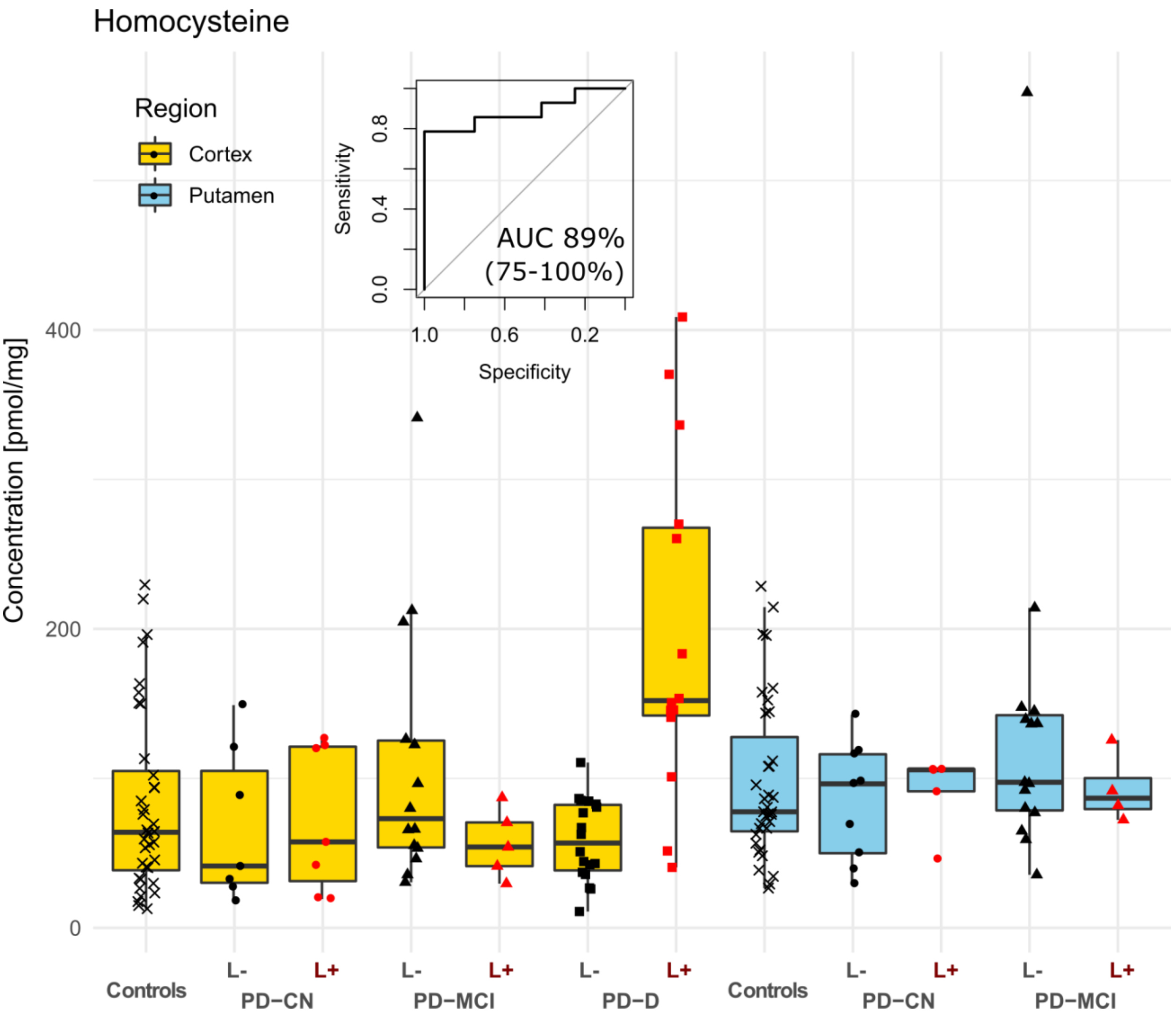

This box plot shows homocysteine concentrations across subject groups (CN – cognitively normal; MCI – with mild cognitive impairment; D – with dementia) in dependence on the acute levodopa medication presence (L+; red points) or physiological range (L-; black points). Note the large levodopa-associated homocysteine increase specific for PD-D group (p = 5e-12, FDR = 2e-10). The ROC curve and AUC refers to the classification of dementia status of PD subjects in L+ state.
