# Supplementary material for: Targeted metabolomic analysis in Parkinson’s disease brain frontal cortex and putamen with relation to cognitive impairment
Source: NPJ Parkinsons Dis. 2023 Jun 3;9:84. doi: 10.1038/s41531-023-00531-y (PMC10239505; doi:10.1038/s41531-023-00531-y)
Supplement: Supplementary file 2 — Reporting Summary [file 41531_2023_531_MOESM2_ESM.pdf]

nature portfolio

Corresponding author(s): DAPR NPJPARKD-01654R2

Last updated by author(s): May 17, 2023

Reporting Summary

Nature Portfolio wishes to improve the reproducibility of the work that we publish. This form provides structure for consistency and transparency in reporting. For further information on Nature Portfolio policies, see our Editorial Policies and the Editorial Policy Checklist.

Statistics

For all statistical analyses, confirm that the following items are present in the figure legend, table legend, main text, or Methods section.

n/a	Confirmed	
☐	☒	The exact sample size (n) for each experimental group/condition, given as a discrete number and unit of measurement
☐	☒	A statement on whether measurements were taken from distinct samples or whether the same sample was measured repeatedly
☐	☒	The statistical test(s) used AND whether they are one- or two-sided<br>*Only common tests should be described solely by name; describe more complex techniques in the Methods section.*
☐	☒	A description of all covariates tested
☐	☒	A description of any assumptions or corrections, such as tests of normality and adjustment for multiple comparisons
☐	☒	A full description of the statistical parameters including central tendency (e.g. means) or other basic estimates (e.g. regression coefficient) AND variation (e.g. standard deviation) or associated estimates of uncertainty (e.g. confidence intervals)
☐	☒	For null hypothesis testing, the test statistic (e.g. F, t, r) with confidence intervals, effect sizes, degrees of freedom and P value noted<br>*Give P values as exact values whenever suitable.*
☒	☐	For Bayesian analysis, information on the choice of priors and Markov chain Monte Carlo settings
☒	☐	For hierarchical and complex designs, identification of the appropriate level for tests and full reporting of outcomes
☒	☐	Estimates of effect sizes (e.g. Cohen's d, Pearson's r), indicating how they were calculated

Our web collection on statistics for biologists contains articles on many of the points above.

Software and code

Policy information about availability of computer code

Data collection	Acquisition: Sciex Analyst v1.6.24; Peak integration: Biocrates MetIDQ Oxygen-DB110-3005
Data analysis	Custom scripts with R v3.6.1 in RStudio v1.2.5033 using R packages as described in the manuscript

For manuscripts utilizing custom algorithms or software that are central to the research but not yet described in published literature, software must be made available to editors and reviewers. We strongly encourage code deposition in a community repository (e.g. GitHub). See the Nature Portfolio guidelines for submitting code & software for further information.

Data

Policy information about availability of data

All manuscripts must include a data availability statement. This statement should provide the following information, where applicable:

- Accession codes, unique identifiers, or web links for publicly available datasets
- A description of any restrictions on data availability
- For clinical datasets or third party data, please ensure that the statement adheres to our policy

The authors declare that the data supporting the findings of this study are available within the manuscript: Measured area ratios along with calculated concentrations and metabolic indicators as well as sociodemographic and clinical information are provided in the Supplementary File 4.

Research involving human participants, their data, or biological material

Policy information about studies with human participants or human data. See also policy information about sex, gender (identity/presentation), and sexual orientation and race, ethnicity and racism.

Reporting on sex and gender	The human samples and sample information were obtained from Banner Sun Health Research Institute brain bank, and the bank reports that the sex of subjects was self-determined with binary classification (male/female), also confirmed by whole body autopsy although not all subjects underwent the biopsy. The reported sex was included as a regression covariate during statistical analysis to control for confounding due to sex-related metabolic differences.
Reporting on race, ethnicity, or other socially relevant groupings	The human samples and sample information were obtained from Banner Sun Health Research Institute brain bank, and the race and ethnicity of subjects were self-determined. We selected samples of subjects reported as White with non-Hispanic or undisclosed ethnicity (see Table 3), achieving a homogeneous sample set. (Only a couple of samples of subjects with other races or Hispanic ethnicity and desired characteristics were available in the bank, which would be difficult to reliably control for the potential confounding effect, leading to increased heterogeneity and lower statistical power, so they were not studied.)
Population characteristics	We analyzed the following main subject groups:<br>1) cognitively normal control subjects without Parkinson's disease (n=36; 61% male; age 82±10; BMI 25±5)<br>2) subjects with Parkinson's disease (n=65; 71% male; age 81±8; BMI 25±7), subdivided by the cognitive status:<br>2a) cognitively normal (n=14; 57% male; age 85±6; BMI 22±4)<br>2b) with mild cognitive impairment (n=19; 74% male; age 82±6; BMI 25±8)<br>2c) with dementia (non-Alzheimer's type) (n=32; 75% male; age 80±5; BMI 27±9) - for this group we had cortex tissue only, no putamen<br><br>Parkinson's disease subjects were treated with levodopa-carbidopa medication and we detected those with the medication presence in the tissue at the time of death, analyzing its immediate effect.<br>No other major pathologies of central nervous system were present.<br>More participant characteristics can be found in Table 3.
Recruitment	The human samples and sample information were obtained from Banner Sun Health Research Institute brain bank, which recruited donors mainly from Phoenix metropolitan area (Arizona, USA). More details can be found under PubMed ID 25619230. We selected tissue samples with appropriate characteristics as defined above.
Ethics oversight	The human samples and sample information were obtained from Banner Sun Health Research Institute brain bank, which is overseen by Banner Sun Health Institutional Review Board.

Note that full information on the approval of the study protocol must also be provided in the manuscript.

Field-specific reporting

Please select the one below that is the best fit for your research. If you are not sure, read the appropriate sections before making your selection.

☒ Life sciences ☐ Behavioural & social sciences ☐ Ecological, evolutionary & environmental sciences

For a reference copy of the document with all sections, see nature.com/documents/nr-reporting-summary-flat.pdf

Life sciences study design

All studies must disclose on these points even when the disclosure is negative.

Sample size	The numbers were based on available tissue in the biobank with required characteristics and post-mortem collection interval range, combined with allocated funding, and exceed the size of most studies with human brain tissue.
Data exclusions	No data were excluded.
Replication	No replication was performed.
Randomization	Samples were randomized across batches with stratification based on the group and sex.
Blinding	Upon randomization, the assignment of samples to groups was kept blinded throughout the tissue processing and biochemical analysis.

Reporting for specific materials, systems and methods

We require information from authors about some types of materials, experimental systems and methods used in many studies. Here, indicate whether each material, system or method listed is relevant to your study. If you are not sure if a list item applies to your research, read the appropriate section before selecting a response.

Materials & experimental systems

n/a	Involved in the study
☒	☐ Antibodies
☒	☐ Eukaryotic cell lines
☒	☐ Palaeontology and archaeology
☒	☐ Animals and other organisms
☒	☐ Clinical data
☒	☐ Dual use research of concern
☒	☐ Plants

Methods

n/a	Involved in the study
☒	☐ ChIP-seq
☒	☐ Flow cytometry
☒	☐ MRI-based neuroimaging
